# Letter to the Editor

**DOI:** 10.1017/S0950268821000819

**Published:** 2021-04-16

**Authors:** Catherine Wright Donnelly, Bronwen E. Percival, Catherine Mead

**Affiliations:** 1 Nutrition and Food Science, University of Vermont, Burlington, Vermont, USA; 2 Specialist Cheesemakers Association, London, UK

To the Editor,

We write to raise several points in response to the paper ‘Microbiological quality of raw drinking milk and unpasteurised dairy products: results from England 2013–2019’ published in *Epidemiology and Infection* in May of 2020.

Our first concern relates to the dataset used as the basis for the preparation of the paper. It is important to recognise sources of bias within the LIMS dataset. The decision to include data from investigations of outbreaks, where many samples are taken from sites experiencing acute difficulties, is questionable, and although the authors maintain that ‘the decision to sample will have been taken on the recognition of risk within [a] manufacturer's premises’, the relatively small dataset, particularly for certain types of cheese, means that these incident data skew the larger picture.

The problem with sampling bias applies more broadly as well. Environmental Health Practitioners do not submit samples from all cheese businesses for testing. As most cheese producers use private contract laboratories for their testing, the officers who submitted the majority of these samples over this 6-year period would have sampled according to perceived need for surveillance, or, for example, as part of the process of deciding whether to approve businesses whose products are not yet legal for sale. Acknowledging this further source of sampling bias is important, but more importantly, care must be taken to avoid using biased data to make assumptions about the entire output of the raw milk cheese industry, or to conclude that raw milk cheeses are ‘a concern for public health’ as is stated in the summary.

When we contacted the authors to raise this point, they suggested that other surveys of cheeses had revealed a similar incidence of indicator organisms and pathogens. Regardless, conclusions drawn from an inappropriate and biased set of data do not add to the weight of scientific fact and should not be published in a peer-reviewed journal.

The authors also call into question industry guidance on acceptable levels of non-toxigenic *Escherichia coli* in raw milk cheese and cite their findings to make the case that more stringent guidelines are appropriate for application to this food. The Specialist Cheesemakers Association guidelines acknowledge that milk produced hygienically, with levels of *E. coli* below the test detection threshold, may give rise to elevated levels in cheese, which undergoes a fermentation step under conditions in which microorganisms are expected to grow. It is, therefore, not surprising to find elevated levels of *E. coli* in some cheeses with a slow acidification profile and high water activity. The Process Hygiene Criteria for Cheese in EU Regulation (EC) 2073/2005 reflect this, with no limit set for levels of *E. coli* in cheese produced from raw milk.

The authors observe that elevated levels of *E. coli* are correlated with the unsatisfactory levels of coagulase-positive *Staphylococcus aureus* in cheese, and argue that decreasing the limit for *E. coli* is therefore indicated.^1^ This conclusion represents a misunderstanding of the biology: these data demonstrate that both organisms, if present, are capable of growth during the production of microbiologically permissive styles of cheese. By confusing correlation with causation, the authors arrive at the incorrect assertion that adopting a lower limit for *E. coli* will be protective against high levels of coagulase-positive *S. aureus*. In fact, these two organisms relate to two completely different aspects of milk production: milking hygiene and animal health. Attempting to solve an animal health problem through increased milking hygiene is an ineffective strategy.

The authors conclude that ‘These data provide evidence for setting criteria for *E. coli* in cheeses made from unpasteurised milk. This group of products is a concern for public health’. However, this conclusion is contradicted by their own statement, ‘Apart from two possible cases of salmonellosis with indistinguishable *Salmonella* newport isolated from a hard cow's milk cheese, analysis of national surveillance databases did not provide any other evidence for disease associated with either consumption of these products, or any other cheeses sample here’. This latter statement strongly supports the premise that current microbiological criteria established for raw milk cheeses are working as intended to protect public health.

In our discussion of how these issues might be avoided in the future, the authors were hesitant to solicit pre-submission feedback from members of industry on the grounds that it could be regarded as introducing commercial bias. However, industry comments from a reputable trade organisation will be based on research undertaken by specialists who frequently have an academic background and are able to provide substantiation and evidence for the views expressed.

Despite our differences, we agree with the authors that there exists a small ‘subgroup of manufacturers where efforts to improve hygiene should be concentrated’. Working with microbiological guidelines that are appropriate to the characteristics of a food and addressing the root causes of risks directly are more effective routes to ensuring a safe food supply than setting unrealistically low limits based on false assumptions and then chastising an entire industry for failing to comply with them.

Sincerely,

Bronwen Percival, on behalf of the Specialist Cheesemakers Association Technical Committee

Catherine Mead, on behalf of the Specialist Cheesemakers Association Executive Committee

Catherine Donnelly PhD, Professor of Nutrition and Food Science, University of Vermont

## Footnotes

1 The results presented here show an association between higher levels of *E. coli* and the presence of unsatisfactory levels of CPS (Table 11). Therefore, the data indicate that the application of more lenient *E. coli* criteria for unpasteurised cheeses, or soft cheeses, for example, cannot be justified in terms of public health, and it is more appropriate to apply the criteria set out in the HPA guidelines [14] to all ready-to-eat foods, including those made from unpasteurised milk.

